# Mapping the road to elimination: a 5-year evaluation of implementation strategies associated with hepatitis C treatment in the veterans health administration

**DOI:** 10.1186/s12913-021-07312-4

**Published:** 2021-12-18

**Authors:** Vera Yakovchenko, Timothy R. Morgan, Matthew J. Chinman, Byron J. Powell, Rachel Gonzalez, Angela Park, Patrick S. Malone, Maggie Chartier, David Ross, Shari S. Rogal

**Affiliations:** 1 Center for Healthcare Organization and Implementation Research, VA Bedford Healthcare System, Bedford, MA USA; 2 Gastroenterology Section, VA Long Beach Healthcare System, Long Beach, CA USA; 3 Center for Health Equity Research and Promotion, VA Pittsburgh Healthcare System, University Drive (151C), Building 30, Pittsburgh, PA 15240 USA; 4 RAND Corporation, Pittsburgh, PA USA; 5 Brown School, Washington University in St. Louis, St. Louis, MO USA; 6 Department of Veterans Affairs, Sierra Pacific Veterans Integrated Service Network, Pharmacy Benefits Management, Mather, CA USA; 7 Department of Veterans Affairs, Office of Healthcare Transformation, Washington, DC USA; 8 Sanford School of Public Policy, Duke University, Durham, NC USA; 9 HIV, Hepatitis, and Related Conditions Programs, Office of Specialty Care Services, Veterans Health Administration, Washington, DC USA; 10 Division of Gastroenterology, Hepatology, and Nutrition, University of Pittsburgh, Pittsburgh, PA USA

**Keywords:** Implementation, Veterans, Diffusion of innovation, Cirrhosis, Liver

## Abstract

**Background:**

While few countries and healthcare systems are on track to meet the World Health Organization’s hepatitis C virus (HCV) elimination goals, the US Veterans Health Administration (VHA) has been a leader in these efforts. We aimed to determine which implementation strategies were associated with successful national viral elimination implementation within the VHA.

**Methods:**

We conducted a five-year, longitudinal cohort study of the VHA Hepatic Innovation Team (HIT) Collaborative between October 2015 and September 2019. Participants from 130 VHA medical centers treating HCV were sent annual electronic surveys about their use of 73 implementation strategies, organized into nine clusters as described by the Expert Recommendations for Implementing Change taxonomy. Descriptive and nonparametric analyses assessed strategy use over time, strategy attribution to the HIT, and strategy associations with site HCV treatment volume and rate of adoption, following the Theory of Diffusion of Innovations.

**Results:**

Between 58 and 109 medical centers provided responses in each year, including 127 (98%) responding at least once, and 54 (42%) responding in all four implementation years. A median of 13–27 strategies were endorsed per year, and 8–36 individual strategies were significantly associated with treatment volume per year. Data warehousing, tailoring, and patient-facing strategies were most commonly endorsed. One strategy—“identify early adopters to learn from their experiences”—was significantly associated with HCV treatment volume in each year. Peak implementation year was associated with revising professional roles, providing local technical assistance, using data warehousing (i.e., dashboard population management), and identifying and preparing champions. Many of the strategies were driven by a national learning collaborative, which was instrumental in successful HCV elimination.

**Conclusions:**

VHA’s tremendous success in rapidly treating nearly all Veterans with HCV can provide a roadmap for other HCV elimination initiatives.

**Supplementary Information:**

The online version contains supplementary material available at 10.1186/s12913-021-07312-4.

## Background

Affecting over 200 million persons globally, hepatitis C virus (HCV) remains the most common chronic bloodborne infection in the United States (US) [1]. The development of highly efficacious, tolerable direct-acting antiviral treatments for hepatitis C virus (HCV) prompted the World Health Organization (WHO) to set ambitious goals for global viral elimination by 2030 [2–4]. However, most countries, including the US, are not on track to meet WHO targets. This is because implementation of new treatments, even of highly efficacious, curative medications, can be challenging due to barriers at the patient, provider, organizational, and systems levels [5]. The field of implementation science, of which this project is an example, emerged to study and address these barriers [6].

Anticipating the coming direct-acting antiviral medications (DAAs), in late 2014, the Veterans Health Administration (VHA) formed the Hepatic Innovation Team (HIT) Collaborative, which consisted of regional, interdisciplinary teams of providers and other stakeholders [7] to support implementation of the new HCV treatment. Organized around Lean principles and quality improvement techniques designed for learning healthcare systems [8–10], the HIT Collaborative helped VHA medical centers (“sites”) employ various implementation strategies, which are “methods or techniques used to enhance the adoption, implementation, and sustainability of a clinical program or practice” [11]. VHA was uniquely positioned to achieve viral elimination, given its unified electronic medical record, nationalized healthcare system, and prioritization of HCV. As such, the VHA far exceeded other healthcare systems [2, 3, 12] by treating nearly 85% of Veterans with known chronic hepatitis C in VHA care [7, 13, 14]. This program evaluation aimed to understand which implementation strategies were influential in achieving that outcome.

“Precision Implementation” describes a growing movement within implementation science to closely consider interacting conditions and context when prescribing and tailoring implementation strategies [15]. Despite advances in naming and specifying implementation strategies, there is a lack of consensus on how to optimally choose strategies throughout the course of a multi-stage implementation [16]. In the context of one specific clinical outcome targeted by a national program, we present a novel approach to collecting and analyzing implementation strategy use longitudinally across the complete lifecycle of an initiative. Specifically, this national evaluation explored: 1) VHA site-level implementation strategy use over time in the largest integrated health care system in the US, 2) associations between strategies and HCV treatment diffusion, and 3) attribution of strategy use to HIT Collaborative support.

## Methods

The HIT Collaborative evaluation was supported by the Department of Veterans Affairs (VA) HIV, Hepatitis, and Related Conditions Program Office in fiscal years (FY) 2015–2019 (October 2014–September 2019). FY15–18 were considered “active implementation” and FY19 was “sustainment.” The VA Pittsburgh Healthcare System IRB reviewed the evaluation protocol and deemed it to be quality improvement per VHA Program Guide 1058.05 [17]. Participation in the evaluation was voluntary and responses to surveys remained confidential.

### Recruitment and data collection

This five-year evaluation included annual surveys of VHA sites treating HCV (*N* = 130, per the VA’s Office of Public Health Definition) [18]. These five years included four active implementation years and one sustainment year. Our survey development process has been previously published [19]. In brief, implementation strategies have historically been hard to define and measure, due to lack of a common and accepted taxonomy. The Evidence-Based Recommendations for Change (ERIC) group of implementation scientists used a review of 205 sources [20] and rigorous modified Delphi Process to name and define 73 implementation strategies [21] (Appendix 1, Additional file 1), followed by concept mapping to place them into nine clusters (e.g., “Provide interactive assistance,” “Train and educate stakeholders”) [22]. With the input of stakeholders, we subsequently converted these strategies into a user-friendly survey format, using parenthetical examples relevant to the clinical topic of HCV [19]. For example, when we inquired about the strategy “Revise professional roles,” we provided the exemplar: (e.g., allow the pharmacist to see and treat patients in the clinic). Given the nascence of implementation strategy studies, all 73 strategies were retained to ensure completeness. We sent the survey to key informants (e.g., gastroenterologists, infectious disease clinicians, HIT members) in each year, encouraging them to complete the survey themselves, obtain input from others or send it to whoever was best positioned to respond [23]. This survey takes respondents 10 min to complete on average.

### Independent variables: implementation strategies and HIT collaborative attribution

Each year, participants reported whether their site used each strategy (yes/no) and, if so, whether the strategy use was attributable to the HIT Collaborative or done independently of their Collaborative involvement. Among the 54 sites that responded in all four active implementation years, strategy dose was defined as the number of years (out of four) that a strategy was endorsed.

### Dependent variables

Our primary effectiveness outcome was site-level HCV treatment, measured as: 1) *treatment volume*, which was defined as the absolute number of patients initiated on a direct-acting antiviral in a fiscal year at each site, and 2) *treatment diffusion peak*, which was defined as the year with the absolute highest number of patients initiating treatment at each site. The use of treatment diffusion peak allowed us to account for the fact that sites had differing numbers of patients in need of treatment. Thus, these two measures collectively provide information about the absolute volume of implementation effort and the rate of implementation.

Treatment diffusion was informed by Rogers’ Diffusion of Innovation Theory [24], which posits that innovation spread is a multi-stage social process led by innovators and early adopters, then accelerated by the majority, and plateaued by the laggards. We classified sites with peak treatment initiation in FY15 as “innovators/early adopters,” in FY16 as “early/late majority,” and FY17–19 as “laggards.” Treatment data were obtained from the national VA Corporate Data Warehouse [25].

### Covariates

To account for possible confounders, several organizational and contextual covariates were collected. At the site level, we collected site complexity, defined by VHA as a composite score incorporating several site-level factors, including patient load and acuity, amount of research funding, the availability of specialty care, and location [26]. For these analyses, we split sites into higher vs. lower complexity. Survey respondent demographic characteristics included staff type, degree, and years in VHA. A summary Team Development Measure (TDM) score and four sub-scores in Communication, Cohesion, Role Clarity, and Goals and Means were obtained at baseline (FY15) and final implementation year (FY18), wherein higher scores on a scale of 0–100 represent higher team functioning [27].

### Analysis

At the site level, we used descriptive statistics to assess the frequency of implementation strategy use and cluster endorsement across the four implementation years and fifth sustainment year and to describe respondent characteristics. We applied Kendall’s τ for non-parametric ordinal correlation analysis to assess strategy use frequency differences between years. Then we examined associations between strategies and HCV treatment initiation at each site, using Spearman’s ρ to determine the association between individual strategies and HCV treatment volume across all responding sites in all years. We then performed Chi-squared tests of independence to determine if treatment diffusion peak was associated with use of each given strategy in each year. We operationalized “local core strategies” as those significantly associated with both treatment volume and treatment diffusion. To examine strategy dose effects, we focused on sites responding in all four active implementation years and conducted correlational analyses between total HCV treatment and total number of years the strategy was used. We also conducted Kruskal-Wallis tests to assess differences in baseline Team Development Measure scores between the three treatment diffusion groups. Finally, we explored how strategies were attributed to the HIT Collaborative over time, operationalizing “core strategies” as those being used by at least 10% of sites in each year and with a positive significant association between treatment volume and attribution to the HIT Collaborative. All analyses were conducted in R 3.6.3 and RStudio 1.2.5033 [28].

## Results

### Respondent characteristics

Across the four years of active HIT Collaborative implementation (FY15–18), and among the 130 sites treating HCV, 127 (98%) responded at least once. Site response rates by year ranged from 62% in FY15 to 84% in FY17 (Table 1). Fifty-four sites (42%) responded in all four years, for a total of 382 responses. In the sustainment year (FY19), 58 sites responded (45%), with 33 (25%) responding in all five years (FY15–19).

**Table 1:** Respondent characteristics

Characteristic	FY15	FY16	FY17	FY18	FY19
	N (%)	N (%)	N (%)	N (%)	N (%)
**Number of sites**	80 (62)	105 (81)	109 (84)	88 (68)	58 (45)
**(of 130 total)**					
**HIT members**	68 (85)	95 (90)	100 (92)	85 (97)	56 (97)
**Years in VA**					
≤ 3	13 (16)	23 (22)	17 (16)	12 (14)	5 (9)
4 to 9	25 (31)	31 (30)	41 (38)	31 (35)	19 (33)
10 to 19	25 (31)	38 (36)	33 (30)	29 (33)	27 (47)
≥ 20	17 (21)	13 (12)	19 (17)	16 (18)	7 (12)
**Specialty**					
GI/Hepatology	33 (4)	42 (40)	40 (37)	34 (39)	32 (55)
Infectious disease	17 (21)	21 (20)	19 (17)	14 (16)	9 (16)
Pharmacy	13 (16)	31 (30)	40 (37)	31 (35)	14 (24)
Primary Care	8 (10)	3 (3)	5 (5)	5 (6)	0 (0)
Other	9 (11)	8 (8)	5 (5)	4 (5)	3 (12)
**Degree**					
PharmD	35 (44)	35 (33)	47 (43)	33 (38)	15 (26)
NP	13 (16)	21 (20)	24 (22)	20 (23)	18 (31)
MD/PA	16 (20)	17 (16)	23 (21)	18 (20)	13 (22)
RN	2 (3)	8 (8)	12 (11)	14 (16)	12 (21)
Other	14 (18)	24 (23)	3 (3)	3 (3)	0 (0)
**Site Complexity**					
1a (most complex)	27 (33)	34 (32)	34 (31)	30 (34)	15 (26)
1b	14 (18)	15 (14)	17 (16)	19 (22)	10 (17)
1c	12 (15)	16 (15)	23 (21)	14 (16)	13 (22)
2	14 (18)	19 (18)	14 (13)	12 (14)	8 (14)
3 (least complex)	12 (15)	21 (20)	21 (19)	13 (15)	12 (21)

*GI* Gastroenterology

### Strategy use over time

The median number of strategies endorsed by site (of 73) were FY15: 24 (interquartile range [IQR] 21), FY16: 27 (IQR 19), FY17: 24 (IQR 24), FY18: 20 (IQR 21), and FY19: 13 (IQR 18). Total strategy endorsement did not differ by respondent specialty, degree, or years in the VHA in any year as determined by Chi-square test.

#### Most popular strategies

The top 10 most frequent strategies were relatively stable over time, with 14 strategies represented in the top 10 across the four active implementation years (Appendix 1, Additional file 1). The three most reported strategies across all active years as determined by consistent presence in the top 10 were: data warehousing techniques (“Integrate clinical records across facilities and organizations to facilitate implementation across systems”), tailoring strategies to deliver HCV care (“Tailor the implementation strategies to address barriers and leverage facilitators that were identified through earlier data collection”), and intervening with patients to promote uptake and adherence to HCV treatment (“Develop strategies with patients to encourage and problem solve around adherence”). In the sustainment year (FY19), the most frequent strategies were again data warehousing (81%) and tailoring strategies to deliver HCV care (69%), with the remainder of strategies having ≤55% endorsement.

#### Strategy use change over time

Some strategies were consistently endorsed over time, whereas others varied over the four years of active implementation and the sustainment year. Twenty-one strategies (29%) across all but one cluster (“Train and educate stakeholders”) significantly varied in endorsement by year (Appendix 1, Additional file 1). The strategy with the widest range of endorsement between years was the “Use mass media to reach large numbers of people” strategy (18–56%). Similarly, “Change the record systems” peaked in FY15 (71%), when the HCV testing clinical reminder was introduced nationally and decreased to 40% by FY18. Between the final active implementation year and sustainment year, the single greatest absolute decline in strategy use was “Changing physical structure and equipment,” from 57 to 29%.

### Individual strategy association with HCV treatment

**Fig. 1:**
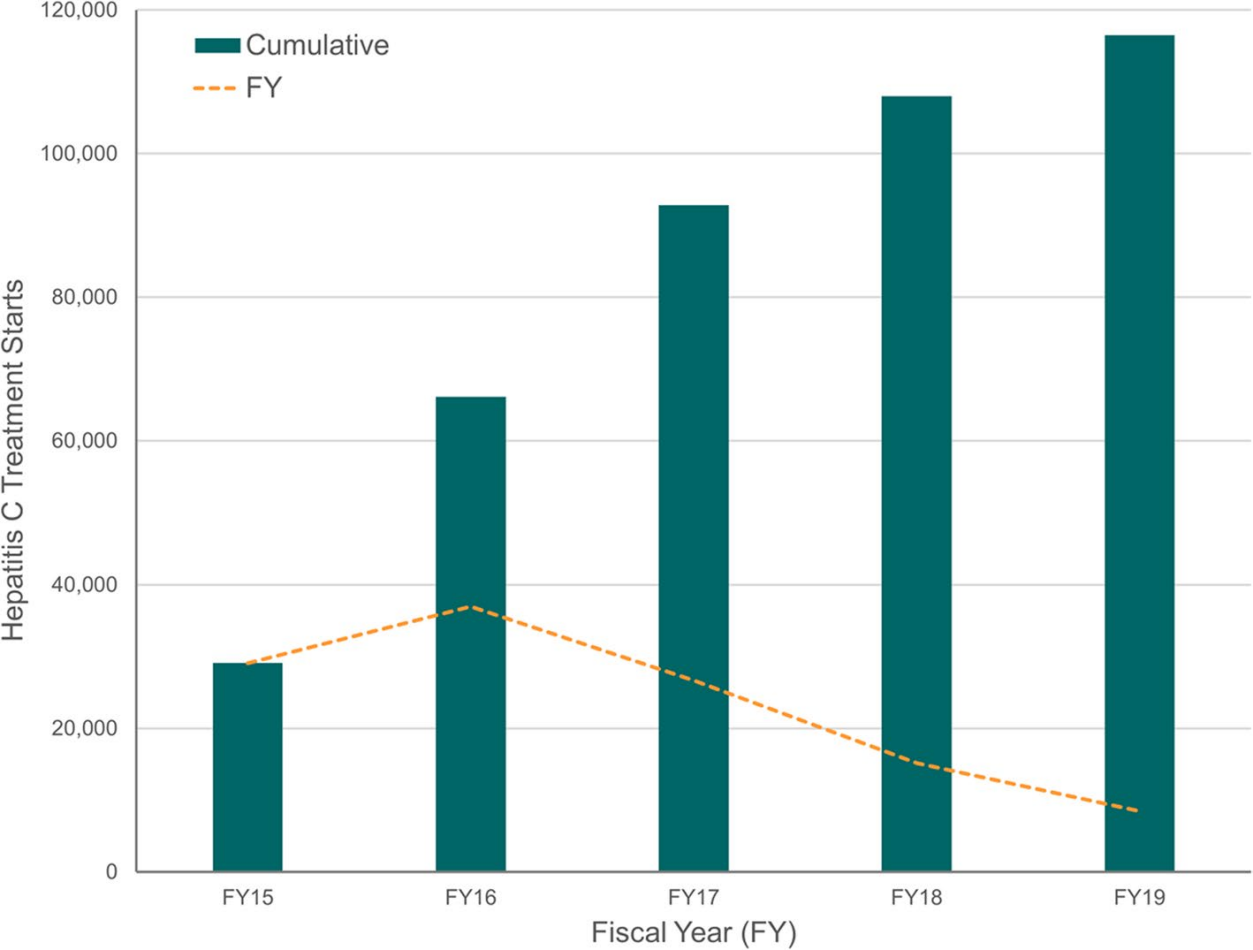
Hepatitis C Treatment in the Veterans Health Administration, FY2015–2019

More than 114,000 Veterans received treatment across all of VHA over the years of study (Fig. 1). Because 98% of sites responded at least once, no comparisons between treatment starts in responding and non-responding sites were conducted over the full evaluation period. Within individual fiscal years, the median number of Veterans with HCV and the median number treated did not vary based on survey response vs. non-response. Respondent specialty, degree and years in the VA was also not associated with treatment volume. The median number of Veterans treated over the study period per site was not significantly different based on the peak treatment year, suggesting that volume of patients was not correlated with rate of treatment.

#### Treatment volume

Over the course of active implementation, 48 of the 73 (66%) strategies were significantly associated with treatment volume in at least one year (Appendix 1, Additional file 1). The number of strategies significantly associated with the number of HCV treatment initiations in each year decreased over time (FY15: 36, FY16: 26, FY17: 11, and FY18: 9). Two strategies were significantly associated with treatment in the sustainment year (FY19): “data warehousing techniques” and “conduct educational meetings.” “Make efforts to identify early adopters to learn from their experiences” was significantly associated with HCV treatment in all four implementation years. Seven strategies were significantly associated with treatment in three years, 17 strategies in two years, and 23 in one. Notably, 25 strategies were never associated with HCV treatment, including seven of the nine strategies from the “Financial” cluster.

#### Strategy dose

We examined strategy dose associations with total HCV treatment among sites that responded in all four years (*n* = 54). Sites could implement strategies from zero to four times in the four years. Among the 73 strategies, Spearman correlation analysis found that 11 strategies had positive and significant dose effects on total treatment volume, meaning the more times a site employed the strategy the higher overall treatment (Appendix 1, Additional file 1). Five of the 11 were strategies from the “Develop stakeholder interrelationships” cluster. The single strongest dose effect was for the “make efforts to identify early adopters to learn from their experiences” strategy (ρ = .41, *p* = .002).

#### Treatment diffusion

Of the 127 sites that responded at least once, 23% were innovators/early adopters, 62% early/late majority, and 15% laggards, according to Rogers’ Diffusion of Innovation typology. Of the 73 strategies, 18 corresponded to peak treatment year as determined by Chi-square tests (Appendix 2, Additional file 1). For some of these strategies a stepwise adoption by diffusion group was evident such that strategy use was highest during peak treatment. For example, “revise professional roles” was used by 68% of innovators/early adopters the strategy in FY15 (vs 14–57% in other years), then 55% of the early/late majority sites in FY16 (vs 35–44% in other years), and finally 69% of laggards in FY17 (vs 20–56% in other years). A similar pattern emerged for “local technical assistance,” “data warehousing techniques,” “identify and prepare champions,” “mandate changes to HCV care,” “conduct small tests of change,” and “provide clinical supervision.”

#### Local core strategies

Based on the strategies identified as significantly associated with both treatment volume and treatment diffusion, we identified 12 “local core strategies” representing all clusters except “Train and educate stakeholders” (Appendix 1, Additional file 1). “Local core strategies” focused on creating new clinical teams, revising professional roles, providing clinical supervision, obtaining implementation support through a champion, offering local technical assistance, acquiring physical equipment, using data warehousing techniques, refining and tailoring HCV care, and preparing patients to be active participants in their HCV care.

#### Team Development Measure

At baseline in FY15, overall Team Development Measure (TDM) scores ranged from 55 to 68 with an average of 61, corresponding to teams being on the cusp of “in place” and “firmly in place.” At the end of implementation (FY18) TDM scores increased to a 65 average (range 59–72) corresponding to reaching “firmly in place.” Among those who responded at both time points, the average change was greatest in Goals and Means (9%) and least in Communication (2%). At baseline, there were no significant differences in overall TDM score by treatment diffusion peak or treatment volume. At follow-up there were team functioning differences by diffusion timing such that innovators, compared to later adopters (“early/late majority” and “laggards” combined), had significantly higher overall scores (*p* = .013) and domain scores in communication (*p* = .019), role clarity (*p* = .006), and goals and means (*p* = .015), and marginally significant higher scores in cohesiveness (*p* = .054).

#### HIT collaborative attribution and core strategies

Across all responses, sites attributed 57% of all strategies to the HIT Collaborative. Clusters differed in attribution to the HIT Collaborative;however, all clusters increased in attribution over time (Table 2). The clusters with the most observed attribution to the HIT Collaborative included “Support Clinicians” (67%), Adapt and “Tailor to Context” (67%), and “Financial” (67%). The greatest increase in attribution between the first and final year of active implementation was in the “Evaluative and Iterative” cluster from 38 to 76%, respectively. We then identified 11 core HIT strategies based on frequency of use and positive association with treatment volume (Appendix 1, Additional file 1). Three of the 11 strategies were from the “Adapt and Tailor to Context” cluster, and another three from the “Evaluative and Iterative” cluster.

**Table 2:** HIT Collaborative Attribution by Strategy Cluster

	Total	FY15	FY16	FY17	FY18	FY19
Overall HIT Attribution	57%	42%	54%	63%	67%	64%
Develop stakeholder relationships	59%	**41%**	**59%**	**66%**	**71%**	66%
Train and educate stakeholders	43%	**27%**	**40%**	**47%**	**55%**	48%
Change infrastructure	63%	**50%**	**55%**	**70%**	80%	77%
Support clinicians	67%	**58%**	**63%**	68%	82%	84%
Provide interactive assistance	54%	**40%**	**58%**	57%	**57%**	70%
Adapt and tailor to context	67%	**59%**	**63%**	76%	**68%**	69%
Engage consumers	39%	**20%**	34%	50%	**47%**	44%
Use eval & iterative strategies	61%	38%	**60%**	72%	76%	64%
Financial strategies	67%	60%	67%	66%	74%	76%

Bold denotes year(s) when cluster had concentration of strategies significantly associated with HCV treatment volume

## Discussion

HCV viral elimination efforts have been a tremendous population health success in VHA. In this longitudinal assessment of implementation strategies, we identified how strategy use, dose, and effectiveness changed over the course of a highly successful national effort, both over time and between sites that were earlier vs. later adopters of direct acting antiviral treatments. We found that specific strategies were consistently associated with peak performance year and delineated the level of implementation by identifying which strategies were driven by the national learning collaborative and which strategies were driven by local context and need. This patterning demonstrates that certain strategies were associated with peak treatment year, indicating that a subset of strategies were prominent regardless of overall implementation phase. Our work advances methodological and conceptual issues relevant to implementation strategies and precision implementation efforts in large healthcare systems.

This implementation study adds to a general understanding of how strategies are employed over the life of an implementation effort [29, 30]. The early strategies included preparatory implementation in the form of both local and central strategies. Early common centralized strategies were driven by support from the HIT Collaborative and focused on creating a structured learning and networking environment, providing resources, making clinical experts available, and developing an HCV population health management dashboard. In contrast, the local strategies were selected to address site-specific context and need, and included building a local team, revising clinical roles, using tools for data monitoring, and engaging patients. These early strategies were followed by data-oriented strategies from the “Evaluative and Iterative”, “Training and Educating Stakeholders,” and “Providing Interactive Assistance” clusters. The sustainment year notably included less of a focus on infrastructure change. The most commonly endorsed strategies in this year were in the “Adapt and Tailor to Context” cluster. Given the nascency of both strategy delineation and evidence about how strategies influence outcomes within implementation science, prescriptive advice about parsimoniously selecting strategies remains an ongoing area of investigation.

Collaboratives are a channel for strategy dissemination and are particularly well suited for large-scale implementation efforts. Because, as has been established, diffusion of innovations is a social process that happens in stages, it may not be surprising that only one strategy—“identify early adopters to learn from their experiences”—was significantly associated with HCV treatment in all implementation years. Accordingly, we found that sites’ peak HCV treatment adoption corresponded to Rogers’ proposed diffusion curve, with innovators/early adopters (24% in our study vs 16% determined by Rogers), early/late majority (61% vs 68% by Rogers), and laggards (15% vs 16% by Rogers). We identified a subset of strategies that had synchronized high frequency use and adoption timing, such that earlier adopters had the highest frequency of use in the first year and laggards had highest frequency of use in the third year. This both illustrates a more linear model of implementation based on deliberate selection of a core set of local strategies and the inherent cascade of influence between adopter types, supporting the premise that innovators/earlier adopters influence the uptake of an innovation for the majority, and who subsequently influence uptake for laggards [29].

Another key finding was that team function and “teamness” were important for early success, while other site characteristics (e.g., size, location, and complexity) were not [31]. We found that earlier adopter sites had higher team functioning related to Cohesion, Communication, and Role Clarity. In accordance with the expected sequence of team development, we observed Goals and Means—the final component of team development—increased over the four implementation years, while earlier aspects of team development were in place earlier in the implementation effort.

Overall, the VHA approach of using a learning collaborative, setting specific goals, increasing capacity to treat, and using data and iterative improvements resulted in efficient and effective treatment. These strategies are consistent with those recommended in the literature and those deployed in the successful viral elimination efforts in Egypt, suggesting that they may be applicable to other implementation efforts [2, 32]. However, in some ways, VHA was uniquely positioned to achieve success. The heightened baseline political and institutional will, infrastructure, and resources facilitated widespread adoption. However, it is notable that, even with these shared aspects of readiness, there was heterogeneity in the degree of treatment. Furthermore, not all sites chose to use the available tools and resources and selection of strategies, allowing us to examine the effects of using these tools and resources. These findings may have limited applicability to more fragmented healthcare systems or where medications are not covered by insurance. However, assuming insurance coverage and administrative motivation, many of these strategies can be used by smaller healthcare systems or networks to promote successful viral elimination.

### Strengths and limitations

This study has several limitations. First, strategies were subject to respondent interpretation, and while the survey inquired about a long list of 73 questions, this list was based on the latest implementation science literature. Moreover, 62–84% of sites recorded responses in each given year, and 42% responded in all years, which is superior to 35% one-time response rates reported in the literature [33]. The dose, intensity, actors, actions and other specifications of strategies are unknown but will be the subject of further inquiry [11]. Likewise, timing and sequencing of and fidelity to strategies within the year are unknown [34]. However, having five years of longitudinal data does allow us to understand the year-to-year shifts. While the relatively small sample size precluded mediation analyses, no site characteristics were related to strategy selection in bivariate analyses. Despite these acknowledged limitations, we present a comprehensive, five-year assessment of implementation strategies across many VHA sites in a highly successful national HCV elimination effort.

## Conclusions

To our knowledge, this is the first study to longitudinally examine implementation strategies in a national implementation effort over a 5-year period. HCV treatment was a remarkable VHA success story and much of the work done to treat Veterans was attributed to the HIT Collaborative support. The interplay of contextual factors, strategy selection, and diffusion time is a novel contribution of this work and underscores the paucity of understanding at the mechanistic level [35, 36]. Measuring implementation strategies nationally allowed us to track the strategies associated with this success and how they evolved over implementation and sustainment.

## Supplementary Information

**Additional file 1: Appendix 1**. Strategy Use and Association with HCV Treatment Over Time. **Appendix 2**. Implementation Strategies Associated with Treatment Diffusion in Specific Patterns.

## Data Availability

These analyses were performed using raw data that are available behind the US Department of Veterans Affairs firewall in a secure research environment. In order to comply with VHA privacy and data security policies and regulatory constraints, only aggregate summary statistics and results of our analyses are permitted to be removed for publication. These restrictions are in place in order to maintain privacy and confidentiality. Access to these data may be granted to persons who are not employees of the VHA following ethical and regulatory approval. Those wishing to access the de-identified data that were used for this analysis may contact Vera Yakovchenko (vera.yakovchenko@va.gov) to discuss the VHA data access approval process.

## Abbreviations

DAA: Direct acting antiviral medication
ERIC: Expert Recommendations for Implementing Change
FY: Fiscal year
HHRC: HIV, Hepatitis, and Related Conditions
HIT: Hepatitis C Innovation Team
HCV: Hepatitis C virus
VA: Department of Veterans Affairs
VHA: Veterans Health Administration
