# Supplementary material for: Mapping the road to elimination: a 5-year evaluation of implementation strategies associated with hepatitis C treatment in the veterans health administration
Source: BMC Health Serv Res. 2021 Dec 18;21:1348. doi: 10.1186/s12913-021-07312-4 (PMC8684191; doi:10.1186/s12913-021-07312-4)
Supplement: Supplementary file 1 — Additional file 1: Appendix 1. Strategy Use and Association with HCV Treatment Over Time. Appendix 2. Implementation Strategies Associated with Treatment Diffusion in Specific Patterns. [file 12913_2021_7312_MOESM1_ESM.pdf]

Appendix 1. Strategy Use and Association with HCV Treatment Over Time

Cluster and Strategy	FY15<br>n=80	FY16<br>n=105	FY17<br>n=109	FY18<br>n=88	FY19<br>n=58	Most Popular<br>P	Timing<br>Δ	Dose<br>E	Diffusion<br>D	Core Strategy<br>H,L
Infrastructure										
• Change physical structure and equipment	***53%***	***51%***	***54%***	57%	29%	P			D	L
• Change the record systems	71%	***57%***	52%	40%	31%	P	Δ			H
• Change the location of clinical service sites	***26%***	***37%***	***39%***	34%	14%					
• Develop a separate organization or group responsible for disseminating HCV care	23%	33%	19%	13%	7%		Δ			
• Mandate changes to HCV care	55%	52%	35%	27%	22%		Δ		D	
• Create or change credentialing and/or licensure standards	29%	30%	17%	16%	17%		Δ			
• Participate in liability reform efforts that make clinicians more willing to deliver the clinical innovation	***4%***	11%	10%	8%	5%					
• Change accreditation or membership requirements	***4%***	1%	2%	0%	0%					
Financial										
• Access new funding	30%	41%	41%	47%	21%					H
• Alter incentive/allowance structures	5%	***10%***	9%	5%	5%				D	L
• Provide financial disincentives for failure to implement or use the clinical innovations	0%	2%	0%	1%	2%					
• Respond to proposals to deliver HCV care	44%	51%	34%	34%	17%		Δ			
• Change billing	11%	14%	10%	5%	5%					
• Place HCV medications on the formulary	70%	69%	61%	45%	28%	P	Δ		D	
• Alter patient fees	0%	0%	0%	0%	0%					

• Use capitated payments	0%	1%	1%	0%	0%					
• Use other payment schemes	***5%***	2%	4%	0%	0%			E		
Support clinicians										
• Create new clinical teams	***46%***	***50%***	39%	36%	21%			E	D	L
• Facilitate the relay of clinical data to providers	***56%***	***68%***	70%	56%	41%	P				
• Revise professional roles	***50%***	***55%***	39%	27%	29%		Δ		D	L
• Develop reminder systems for clinicians	34%	44%	34%	43%	29%				D	
• Develop resource sharing agreements	***26%***	35%	28%	25%	21%					H
Provide interactive assistance										
• Use outside assistance often called “facilitation”	8%	12%	24%	20%	12%		Δ			
• Provide technical assistance	***15%***	25%	25%	***19%***	12%				D	L
• Provide clinical supervision	***44%***	***48%***	35%	32%	28%				D	L
• Use a centralized system to deliver facilitation	***28%***	28%	25%	***24%***	24%			E		H
Adapt and tailor to the context										
• Use data experts to manage HCV data	***58%***	***70%***	69%	***44%***	47%	P	Δ	E		H
• Use data warehousing techniques	***85%***	91%	92%	86%	***81%***	P			D	L
• Tailor strategies to deliver HCV care	***63%***	81%	73%	70%	69%	P	Δ		D	H,L
• Promote adaptability	***55%***	75%	70%	61%	52%	P	Δ			H
Train and educate stakeholders										
• Conduct educational meetings	***51%***	64%	68%	53%	***41%***	P				
• Have an expert in HCV care meet with providers to educate them	***41%***	53%	51%	48%	33%					
• Provide ongoing HCV training	***49%***	60%	54%	***44%***	34%			E		
• Facilitate the formation of groups of providers and fostered a collaborative learning environment	***44%***	***43%***	***38%***	31%	26%					
• Developed formal educational materials	39%	35%	39%	30%	12%					

• Distribute educational materials	55%	***55%***	58%	52%	33%					
• Provide ongoing consultation with one or more HCV treatment experts	58%	71%	59%	56%	55%	P				
• Train designated clinicians to train others	20%	26%	26%	20%	17%				D	
• Vary the information delivery methods to cater to different learning styles when presenting new information	***36%***	36%	31%	32%	26%					
• Give providers opportunities to shadow other experts in HCV	33%	22%	***20%***	***23%***	19%					
• Use educational institutions to train clinicians	***11%***	***15%***	12%	19%	14%			E		
Develop stakeholder interrelationships										
• Build a local coalition/team to address challenges	***53%***	***53%***	***38%***	27%	26%		Δ		D	L
• Conduct local consensus discussions	***48%***	***54%***	***50%***	32%	22%		Δ			
• Obtain formal written commitments from key partners that state what they will do to implement HCV care	4%	4%	6%	7%	0%			E		
• Recruit, designate, and/or train leaders	***26%***	***23%***	18%	14%	10%					
• Inform local opinion leaders about advances in HCV care	***49%***	46%	***40%***	***32%***	29%			E		
• Share the knowledge gained from quality improvement efforts with other sites outside your medical center	***38%***	57%	***54%***	36%	31%		Δ	E		H
• Identify and prepare champions	***50%***	52%	***50%***	40%	38%				D	L
• Organize support teams of clinicians, give them time to share the lessons learned and support	26%	***32%***	32%	***18%***	17%					

one another’s learning										
• Use advisory boards and interdisciplinary workgroups to provide input into HCV policies and elicit recommendations	26%	22%	21%	14%	10%					
• Seek the guidance of experts in implementation	44%	50%	44%	35%	33%					
• Build on existing high-quality working relationships and networks to promote information sharing and problem solving	***61%***	71%	62%	58%	47%	P				
• Use modeling or simulated change	***13%***	***15%***	11%	10%	5%					
• Partner with a university to share ideas	***14%***	11%	13%	17%	7%			E		
• Make efforts to identify early adopters to learn from their experiences	***16%***	***24%***	***21%***	***15%***	12%			E		
• Visit other sites outside your medical center to try to learn from their experiences	***15%***	20%	18%	18%	16%					
• Develop an implementation glossary	3%	6%	6%	5%	2%					
• Involve executive boards	23%	***33%***	29%	17%	14%					
Use evaluative and iterative strategies										
• Assess for readiness and identify barriers and facilitators to change	26%	***30%***	26%	22%	17%					
• Conduct a local needs assessment	45%	43%	39%	24%	24%		Δ			H
• Develop a formal implementation blueprint	34%	***36%***	29%	22%	14%					H
• Start with small pilot studies and then scale them up	23%	25%	23%	17%	10%				D	H
• Collect and summarize clinical performance data and give it to clinicians and	***21%***	***26%***	30%	19%	19%					

administrators to implement changes in a cyclical fashion using small tests of change before making system-wide changes										
• Conduct small tests of change, measured outcomes, and then refine these tests	19%	***21%***	***27%***	18%	10%				D	L
• Develop and use tools for quality monitoring	41%	32%	28%	22%	16%		Δ		D	
• Develop and organize systems that monitor clinical processes and/or outcomes for the purpose of quality assurance and improvement	30%	***28%***	24%	13%	12%		Δ			
• Intentionally examine the efforts to promote HCV care	61%	***69%***	61%	52%	43%	P				
• Develop strategies to obtain and use patient and family feedback	20%	***20%***	11%	13%	10%					
Engage consumers										
• Involve patients/consumers and family members	50%	61%	50%	44%	34%					
• Engage in efforts to prepare patients to be active participants in HCV care	***63%***	57%	50%	43%	34%	P	Δ		D	L
• Intervene with patients/consumers to promote uptake and adherence to HCV treatment	71%	79%	68%	60%	50%	P	Δ			
• Use mass media to reach large numbers of people	18%	36%	56%	34%	12%		Δ			
• Promote demand for HCV care among patients through any other means	***40%***	52%	59%	***42%***	31%		Δ			

Bold italicized denotes p<.05 for association between strategy and HCV treatment volume (rho range=.19-.41)
P = Most popular strategies across active implementation years (n=14)
Δ = Strategy use changed over time (n=21)
E = Strategy has dose effect on HCV treatment volume (n=11)
D = Strategy differed in use by Treatment Diffusion group in at least one of the four implementation years (n=18)

H = Core HIT Strategies (n=11)
L = Local Core Strategies (n=11)

Appendix 2. Implementation Strategies Associated with Treatment Diffusion in Specific Patterns

Strategy	FY15	FY16	FY17	FY18	FY19
Change physical structure and equipment					
Innovator	63%	26%	39%	**71%**	7%
Majority	48%	**64%**	53%	50%	38%
Laggard	55%	38%	**81%**	60%	36%
Mandate changes to HCV care					
Innovator	**53%**	52%	35%	38%	0%
Majority	**58%**	53%	31%	25%	28%
Laggard	45%	**50%**	50%	20%	36%
Alter incentive/allowance structures					
Innovator	**16%**	9%	9%	0%	0%
Majority	0%	8%	**10%**	8%	9%
Laggard	9%	**19%**	6%	0%	0%
Place HCV medications on the formulary					
Innovator	68%	**83%**	61%	43%	7%
Majority	**64%**	64%	64%	44%	28%
Laggard	**100%**	69%	50%	53%	55%
Create new clinical teams					
Innovator	58%	**61%**	57%	33%	7%
Majority	**44%**	47%	30%	38%	28%
Laggard	36%	44%	**56%**	33%	18%
Revise professional roles					
Innovator	**68%**	57%	26%	14%	7%
Majority	44%	**55%**	36%	35%	34%
Laggard	45%	56%	**69%**	20%	45%
Develop reminder systems for clinicians					
Innovator	21%	30%	39%	**43%**	13%
Majority	32%	**48%**	33%	44%	44%
Laggard	**64%**	44%	31%	40%	9%
Have someone from inside the clinic tasked with “local technical assistance”					
Innovator	**26%**	13%	26%	14%	13%
Majority	14%	**30%**	19%	25%	16%
Laggard	0%	19%	**50%**	7%	0%
Provide clinical supervision					
Innovator	**47%**	30%	39%	14%	20%
Majority	44%	**53%**	36%	42%	34%
Laggard	36%	**50%**	25%	20%	18%
Use data warehousing techniques					
Innovator	**89%**	78%	87%	86%	80%
Majority	84%	**95%**	93%	88%	81%

Laggard	82%	94%	**94%**	80%	82%
Tailor strategies to deliver HCV care					
Innovator	68%	74%	**87%**	81%	53%
Majority	56%	**86%**	67%	65%	84%
Laggard	**82%**	69%	81%	73%	45%
Train designated clinicians to train others					
Innovator	21%	17%	**22%**	14%	7%
Majority	22%	**33%**	29%	25%	25%
Laggard	9%	6%	**19%**	13%	9%
Build a local coalition/team to address challenges					
Innovator	53%	**65%**	30%	33%	13%
Majority	**54%**	53%	31%	31%	31%
Laggard	45%	38%	**75%**	7%	27%
Identify and prepare champions					
Innovator	**58%**	39%	35%	43%	47%
Majority	52%	**58%**	49%	38%	38%
Laggard	27%	50%	**75%**	40%	27%
Start with small pilot studies and then scale them up					
Innovator	21%	9%	**22%**	14%	7%
Majority	24%	**26%**	23%	19%	9%
Laggard	18%	**44%**	25%	13%	18%
Conduct small tests of change, measure outcomes, and then refine these tests					
Innovator	**21%**	13%	13%	14%	7%
Majority	20%	21%	**26%**	19%	13%
Laggard	9%	31%	**50%**	20%	9%
Develop and use tools for quality monitoring					
Innovator	**42%**	17%	30%	14%	7%
Majority	**42%**	38%	21%	21%	25%
Laggard	36%	31%	**56%**	33%	0%
Engage in efforts to prepare patients to be active participants in HCV care					
Innovator	**84%**	52%	57%	38%	33%
Majority	52%	**59%**	46%	44%	38%
Laggard	**73%**	56%	56%	47%	27%

Bold denotes highest use of the strategy per diffusion group
